# Bcl-XL is qualitatively different from and ten times more effective than Bcl-2 when expressed in a breast cancer cell line

**DOI:** 10.1186/1471-2407-6-213

**Published:** 2006-08-23

**Authors:** Aline A Fiebig, Weijia Zhu, Catherine Hollerbach, Brian Leber, David W Andrews

**Affiliations:** 1 Department of Biochemistry and Biomedical Sciences, McMaster University, Hamilton, Canada; 2 Departments of Medicine and Biochemistry and BiomedicalSciences, McMaster University, Hamilton, Canada

## Abstract

**Background:**

Bcl-2 and Bcl-XL are anti-apoptotic paralogues that inhibit apoptosis elicited by a wide variety of stimuli, and play critical roles in cancer development and resistance to treatment. Many clinical studies have indicated that expression of these anti-apoptotic proteins in tumours is associated with poor prognosis. It has therefore been assumed that in cells the essential difference between Bcl-2 and Bcl-XL involves regulation of expression and that they are otherwise functionally similar. To examine this issue, we have compared the function of the proteins and of mutants of Bcl-2 and Bcl-XL specifically targeted to different subcellular sites.

**Methods:**

We generated clones of the human breast cancer line MCF-7 stably expressing known amounts of Bcl-2, or Bcl-XL as determined by quantitative immunoblotting. Clones expressing equivalent amounts of wild-type and mutants of Bcl-2 and Bcl-XL with subcellular localization restricted to the cytoplasm, endoplasmic reticulum or outer mitochondrial membrane were studied in both MCF-7 and Rat-1 fibroblasts. In MCF-7 cells we measured the functional activities of these proteins in preventing apoptosis induced by four different agents (doxorubicin, ceramide, thapsigargin, TNF-α). Etoposide and low serum were used to compare the effect of Bcl-2, Bcl-XL and mutants located at the endoplasmic reticulum on induction of apoptosis in fibroblasts.

**Results:**

We noted both qualitative and quantitative differences in the functional activity of these two anti-apoptotic proteins in cells: Bcl-2 localized to the endoplasmic reticulum inhibits apoptosis induced by ceramide and thapsigargin but not by doxorubicin or TNFα, while Bcl-XL at the endoplasmic reticulum is active against all four drugs. In fibroblasts Bcl-2 localized to the ER did not prevent cell death due to etoposide whereas Bcl-XL in the same location did. Finally in MCF-7 cells, Bcl-XL is approximately ten times more active than Bcl-2 in repressing apoptosis induced by doxorubicin. This difference can be manifest as a large difference in clonal survival.

**Conclusion:**

When examined in the same cellular context, Bcl-2 and Bcl-XL differ substantially in the potency with which they inhibit apoptosis, mediated in part by differences in the inhibition of specific subcellular pathways.

## Background

Apoptosis is a critical process that is dysregulated in tumourigenesis [1]. Bcl-2 was the prototypic anti-apoptotic protein, and Bcl-XL was the first protein discovered with similar function [2]. Since then the Bcl-2 family has expanded to include more than 6 anti-apoptotic and many pro-apoptotic members [3]. Bcl-2 and Bcl-XL display 43 % amino acid identity, share regions of sequence similarity [4,5] as well as a C-terminal hydrophobic region required for membrane localization [2], and represent the most recent additions to the Bcl-2 family [6]. Bcl-2 and Bcl-XL appear to function in the same apoptotic pathway [7], and both confer resistance to multiple chemotherapy agents when tested in experimental systems. Over-expression of either protein is usually associated with poor prognosis in many human cancers (reviewed in 8). However, in some cancer types multiple anti-apoptotic proteins are expressed [9], and have opposite effects on prognosis [10-12] indicating that there may be subtle, but clinically and biologically relevant functional differences between family members. Experiments in mice with deletion of individual anti-apoptotic genes indicate that the phenotypes are not identical [13]. However, it is generally accepted that this is due to expression in different tissues or in the same tissue but at different times rather than being a consequence of differences in the potency or mechanism of action of the different anti-apoptotic proteins.

The mechanisms of action of Bcl-2 and Bcl-XL are complex, with many postulated interactions with other proteins, and the role of any single interaction in the final phenotype at the cellular level remains ill-defined. Bcl-2 is located at the mitochondrion, endoplasmic reticulum (ER) and the nuclear envelope [14,15]. Bcl-XL resides in the nuclear envelope, extra-nuclear membranes, including the mitochondrion but also cytosol [16,17]. Bcl-2 is targeted to membranes by a carboxyl-terminal tail-anchor [15], and by replacing the tail-anchor with heterologous sequences specific for insertion into either ER or mitochondria, we have created fusion proteins targeted to individual organelles [18]. These targeted mutants defined distinct but overlapping Bcl-2 regulated apoptosis pathways at individual organelles [18-22]. Here we have created similar mutants of Bcl-XL to compare organelle specific inhibition of apoptosis by Bcl-2 and Bcl-XL.

The human breast cancer cell line MCF-7 transfected with plasmids expressing either Bcl-2 or Bcl-XL is an excellent system in which to examine the differences between these two proteins as the cells do not express detectable Bcl-XL, and endogenous Bcl-2 can be drastically reduced by growth in estrogen depleted medium [23]. Therefore, the background due to endogenous anti-apoptotic proteins is minimal, and the effects of exogenously expressed proteins can be monitored. As adherent cells, they can be studied by immunofluorescence microscopy to determine the location of organelle-targeted mutants. MCF-7 cells lack caspase-3 due to a genomic deletion [24], potentially decreasing feedback activation of other caspases, simplifying the analysis of individual apoptotic pathways [25]. Finally, most human breast cancers originate in epithelial cells that express Bcl-2 or Bcl-XL [26-28]. Clinical studies have demonstrated that increased levels of Bcl-XL in breast carcinoma are associated with a poor outcome [12]. Conversely and paradoxically, Bcl-2 expression confers a better prognosis than lack of expression [10,11]. However, the association of Bcl-2 with expression of the estrogen receptor (a strong independent predictor of good outcome) has confounded these studies.

To examine different apoptosis pathways in cells expressing Bcl-2, Bcl-XL or one of the targeted mutants, we examined the response of MCF-7 cells to several stimuli that differentially engage organelle specific pathways of apoptosis [29,30]. To determine how general the qualitative differences between Bcl-2 and Bcl-XL are we also assessed the function of the wild-type proteins and targeted mutants of Bcl-2 and Bcl-XL in a Rat-1 fibroblast cell line used previously to demonstrate differences in the regulation of apoptosis at ER and mitochondria [18,29,30].

Doxorubicin was selected to examine the differences between the various cell clones quantitatively, as this drug is used extensively in treating breast cancer patients and it is well characterized as an apoptotic stimulus for MCF-7 cells [23]. Thus, quantitatively assaying doxorubicin-induced apoptosis, both by monitoring the cleavage of PARP and by assessing clonogenic survival in the estrogen receptor positive MCF-7 line represents an excellent cellular context in which to measure and analyze differences between Bcl-2 and Bcl-XL. Unexpectedly, our results demonstrate that Bcl-2 and Bcl-XL differ substantially in the potency with which they inhibit apoptosis, mediated in part by differences in the inhibition of specific subcellular pathways. These data suggest caution in interpreting expression alone as directly proportional to functional activity in clinical samples, and clearly show that Bcl-2 and Bcl-XL are functionally distinct.

## Methods

### Plasmids and recombinant proteins

Plasmids encoding targeted Bcl-2 mutants (Bcl2-acta and Bcl2-cb5) have been previously described [18]. A similar strategy was used for Bcl-XL: the cDNA encoding amino acids 1 – 210 of Bcl-XL was fused either to the sequence encoding the carboxyl-terminus of ActA or the cytochrome b5 hydrophobic tail for mitochondrial or endoplasmic reticulum targeting, respectively. Bcl-XL lacking the carboxyl-terminal sequence was generated by inserting a stop codon after nucleotide 630.

To purify Bcl-XL, DH5α cells were transformed with plasmid encoding a Bcl-XL-intein fusion protein. The fusion protein was expressed and bound to a chitin column; after washing the column, cleavage of the fusion protein was induced with 30 mM DTT. Recombinant glutathione-*S*-transferase (GST)-Bcl-2ΔTM [13] was purified on a glutathione column.

### Immunofluorescence

MCF-7 cells were analyzed by immunofluorescence as previously described [18], using rabbit anti-Bcl-XL antisera followed by either a monoclonal antibody to the ER protein calreticulin, or to an inner mitochondrial membrane protein (2G2, ExAlpha Biologicals). Where indicated, FITC was coupled directly to the primary antibody. FITC and rhodamine donkey anti-rabbit and rhodamine donkey anti-mouse were used as secondary antibodies. Cells were analyzed using a Zeiss confocal microscope and associated software (Carl Zeiss LSM510). To assess apoptosis in rat fibroblasts the cells were stained with Hoescht 33342 and Annexin V coupled to Alexa Fluor594 according to specifications of the manufacturer (Molecular Probes) and viewed by epifluorescence microscopy.

### Cell culture

Stable transfectants of MCF-7 and Rat-1-Myc^ERTAM ^(referred to here as Rat-1) cell lines generated using Geneporter (Gene Therapy Systems Inc) were analyzed for expression by quantitative immunoblotting. Apoptosis was induced in Rat-1 cells using etoposide or serum starvation as described previously [29,30]. To reduce expression of endogenous Bcl-2 in the MCF-7 cells the cells were incubated in phenol red minus αMEM with 10 % charcoal filtered FBS for 6 days prior to each experiment. MCF-7 cells were washed with PBS and incubated in the same medium containing: doxorubicin (at the specified concentrations, for 24 hours), thapsigargin (400 nM, for 60 hours), ceramide (70 μM, for 20 hours), or TNFα and cycloheximide (29 ng/ml and 10 ng/ml, respectively for 30 hours). Floating and adherent cells were harvested and pelleted by centrifugation, the cell pellet washed twice in PBS and resuspended in SDS lysis buffer (10 mM Tris-HCl, pH 7.5; 10 mM NaCl; 1 % SDS and protease inhibitors). An aliquot was removed for protein concentration determination by BCA protein assay (Pierce), and the rest of the cell lysate was diluted 1:1 with hot 4 % SDS, 0.1 M Tris-HCl pH 8.9, 2 mM EDTA, 0.1 % bromophenol blue, 20 % glycerol and 0.25 M DTT, and stored at -80°C.

### Quantitative immunoblotting

Cell lysates were processed as previously described [18]. The primary antibodies, source and dilutions used for immunoblotting were as follows: Bcl-2 (sheep polyclonal generated using GST-Bcl-2Δt; our laboratory; 1:10,000), Bcl-XL (rabbit polyclonal generated against Bcl-XL; our laboratory; 1:10,000), PARP (C-2-10, Biomol; 1:20,000), caspases 7, 8 and 9 (ExAlpha Biologicals; 1:4,000). Actin blots to ensure equivalent loading were developed with clone C4 (ICN) diluted at 1:80,000. HRP linked secondary antibodies (Jackson Laboratories) donkey anti-rabbit and donkey anti-mouse were used at dilutions of 1:10,000 and 1:80,000, respectively. To measure expression, the amount of exogenously expressed protein/microgram total protein was determined by quantitative immunoblotting of lysates from each MCF-7 clone. Quantification was performed using a standard curve of defined amounts of recombinant Bcl-2 or Bcl-XL from 3–6 blots, using amounts of protein that were in the linear detection range of enhanced chemiluminescence. The immunoblots were analyzed using the Kodak Image Station system (440CF). A linear regression analysis was performed on the net intensities of the standards and blots in which the lines of best fit with R^2 ^values of ≥ 0.95 were analyzed. To identify appropriate clones of MCF-7 cells for further study, more than thirty were screened. The concentration of Bcl-2 or Bcl-XL in each MCF-7 clone used for further experiments was assayed at least three times and showed low inter-assay variability (<10%).

### Measurement of cell death

During apoptosis PARP is cleaved into 24 and 89 kDa fragments by caspase-7 in MCF-7 cells [31] and can be degraded by lysosomal proteases to 62 and 55 kDa or 74 and 42 kDa fragments [32]. The C-2-10 monoclonal antibody recognizes full length and 89 kDa caspase cleaved PARP, but does not recognize the cleavage products of other proteases. Using this and other commercially available antibodies it is only possible to visualize the full length protein, 89 and 24 kDa fragments. Therefore, to assess PARP cleavage by caspase 7 as well as degradation we measured the decrease in the amount of the full length PARP. In each experiment the amount of actin in the samples was also recorded. If the actin blots indicated uneven loading, transfer or development of the blots then the corresponding PARP data was not used. Doxorubicin induced degradation of actin was detected only at drug concentrations higher than 100 μM. Clonogenic survival after doxorubicin exposure was performed as previously described [33].

## Results

Although generally regarded as mechanistically similar, the effectiveness of Bcl-2 and Bcl-XL has not been compared in a single defined cellular context. Such a comparison is difficult because both proteins inhibit multiple different pathways for apoptosis within a single cell line. However, for a defined set of stimuli it is possible to compare Bcl-XL and Bcl-2 in the same context by measuring a common event that occurs at or beyond a point of convergence that is common to multiple cell death pathways. Thus, the first step in comparing Bcl-2 and Bcl-XL is identification of suitable downstream events that can be assayed.

### Bcl-2 and Bcl-XL inhibit apoptosis induced by a variety of agents in MCF-7 cells

**Figure 1:**
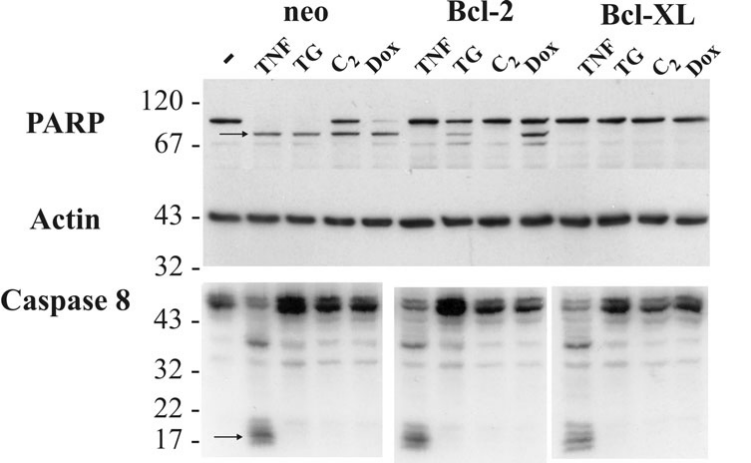
**Prevention of apoptosis by Bcl-2 and Bcl-XL in MCF-7 cells. **Cell lysates (20 μg of protein) of vector control (neo) and transfected MCF-7 cells expressing Bcl-2 or Bcl-XL (as indicated) were separated by SDS-PAGE, electroblotted and visualized using antibodies for PARP, actin or caspase 8 as indicated. Untreated vector transfected cells (-) as a control for expression level in untreated cells. Expression of Bcl-2 and Bcl-XL does not change the amount of PARP in untreated cells. Cells were exposed to 29 ng/ml TNFα and 10 ng/ml cycloheximide (TNF) (30 h), 400 nM thapsigargin (TG) (60 h), 70 μMC_2_-ceramide (C_2_) (20 h), or 10 μM doxorubicin (Dox) (24 h) as indicated above the lanes. The migration of the cleavage products of PARP and caspase 8 are indicated to the left of the bands by arrows. The migration positions of molecular weight markers are indicated in kDa to the left of the panels.

The drugs used to induce apoptosis are known to promote apoptosis via different pathways: experiments with MCF-7 and Rat-1 fibroblasts suggested that TG and ceramide induce apoptosis at the endoplasmic reticulum upstream of events at mitochondria [29,30]. The initial target site of thapsigargin (TG) is the ER Ca^++ ^pump SERCA2 [34]. C_2 _ceramide is a soluble version of a signalling molecule generated at the ER [35]. Mechanisms regulated at the endoplasmic reticulum are not expected to be involved in doxorubicin induced apoptosis [30] as it inhibits topoisomerase II and generates free radicals [36]. TNFα activates caspase 8 to cleave Bid which in turn triggers Bax/Bak to permeabilize mitochondria [37,38]. To determine an appropriate way to compare apoptosis elicited by these different mechanisms in MCF-7 cells, we examined cells treated with the different agents for cleavage of caspases and PARP as well as using Hoescht and Annexin V staining. Apoptosis was measured at a variety of concentrations; however, in Figure 1 a single concentration and time point are shown for each drug to facilitate visual comparison. The dose and time were selected to maximize the difference between control and Bcl-2/Bcl-XL expressing cells.

In vector transfected control cells all four agents induced apoptosis, as assessed by the cleavage of PARP (Figure 1), demonstrating that the effects on PARP occur downstream of a point of convergence. In contrast, only TNFα led to cleavage of caspase 8 (Figure 1). Furthermore, the absence of caspase 8 cleavage with TG, ceramide and doxorubicin establishes that caspase 8 cleavage cannot be used to compare induction of apoptosis by TNFα with the other agents.

MCF-7 cell lines expressing Bcl-XL and Bcl-2 were protected against apoptosis as judged by PARP cleavage induced by TNFα, TG, ceramide and doxorubicin compared to vector transfected (neo), control cells (Figure 1). However, neither Bcl-2 nor Bcl-XL prevented TNFα induced cleavage of caspase 8, indicating that both proteins act downstream of activated caspase 8. Thus both Bcl-2 and Bcl-XL inhibit downstream events elicited by four agents that initiate cell death via different pathways. To compare drugs that induce DNA damage (doxorubicin) and those that act at the ER and do not cause direct changes at the nucleus (TG) methods such as TUNEL staining or chromatin condensation are not optimal. For similar reasons, assessing cleavage of other caspases proved impractical for comparing both a variety of drugs and cells expressing Bcl-2 and Bcl-XL (data not shown).

The data in Figure 1 suggest that for MCF-7 cells, assaying PARP cleavage can be used to determine whether organelle targeted mutants of Bcl-2 and Bcl-XL show the same pattern of protection against the mechanistically distinct apoptosis pathways activated by these four different drugs. While not useful for all of the drugs studied here, we found that chromatin condensation (assessed by Hoescht staining) and externalization of phosphatidylserine (assessed by Annexin V labelling) were useful to compare some subsets of apoptotic agonists particularly in Rat-1 cells (see below).

### Expression and subcellular localization of Bcl-2 and Bcl-XL mutants in MCF-7 cells

Bcl-2 and Bcl-XL inhibit multiple different pathways of apoptosis. To examine the inhibition of multiple spatially localized apoptosis pathways, we substituted the tail-anchor from Bcl-XL with one specific for ER (cb5) or mitochondria (acta), as done previously with Bcl-2 [18]. Chimaeras of Bcl-2 or Bcl-XL with the cb5 or acta tail anchors are referred to as Bcl2-cb5 or BclX-cb5, and Bcl2-acta or BclX-acta, respectively. Chimaeras of Bcl-2 and Bcl-XL without a tail-anchor are referred to as Bcl2-Δt and BclX-Δt, respectively. MCF-7 clones are referred to by the proteins expressed. Quantitative western blotting was used to select MCF-7 clones in which expression of the chimaeras was equivalent to that of the wild-type proteins.

**Figure 2:**
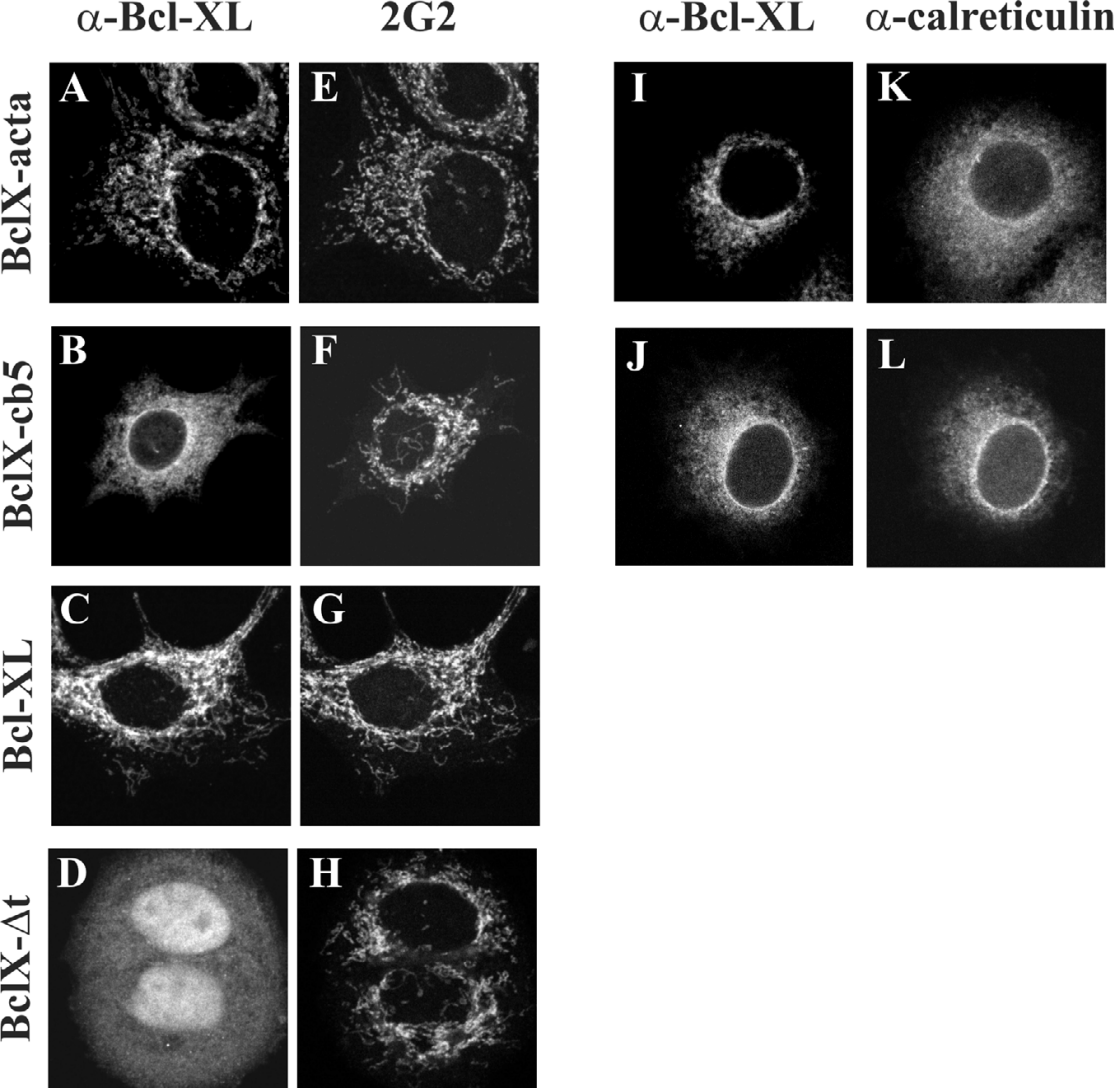
**Subcellular localization of Bcl-XL, BclX-acta, BclX-cb5 and BclX-Δt. **Panels **A-D**, cells stained with an affinity purified polyclonal antiserum to full length Bcl-XL, and visualized with a fluorescein-conjugated donkey anti-rabbit antibody. Panels **E-H**, the corresponding images of the same cells in **A-D **stained with a monoclonal antibody to a mitochondrial inner membrane protein (2G2) and visualized with rhodamine-conjugated goat anti-mouse antibody. Panels **I-J**, cells stained with an affinity purified polyclonal antiserum to full length Bcl-XL conjugated directly to fluorescein and **K-L**, the corresponding images of the same cells stained with a polyclonal antibody to calreticulin. The protein expressed in each cell line is indicated to the left of the panels. The width of each image is 50 microns.

Bcl2-cb5 and Bcl2-acta are localized to ER and mitochondria, respectively in several different cell lines [18-21,30]. However, localization of BclX-cb5 and BclX-acta has not been examined previously. We therefore stained MCF-7 cells expressing targeted mutants with polyclonal antisera to either Bcl-XL (Figure 2, panels A-D, I-J) or Bcl-2 (not shown), and examined them by indirect immunofluorescence using confocal microscopy. Cells were co-stained with antibodies recognizing either an inner mitochondrial membrane protein (Figure 2, 2G2, panels *E-H*) or an ER protein (anti-calreticulin, Figure 2, panels *K-L*). As expected, both BclX-acta and Bcl2-acta (data not shown) are targeted to mitochondria in MCF-7 cells, as indicated by the extensive co-localization observed with 2G2 (Figure 2, panels *A, E*), but not with the endoplasmic reticulum chaperone calreticulin (Figure 2, panels *I, K*). The distribution of wild-type Bcl-XL co-localized with 2G2 indicating that in MCF-7 cells the Bcl-XL expressed exogenously is located primarily at mitochondria (Figure 2, panels *C, G*). Wild-type Bcl-2 had a similar distribution in MCF-7 cells (data not shown). By contrast, BclX-cb5 (Figure 2, panels *B, F*) and Bcl2-cb5 (data not shown) do not localize to mitochondria, but instead exhibited a net-like cytoplasmic staining that co-localized with calreticulin (Figure 2 panels *J, L*). Bcl-XL without a tail-anchor (BclX-Δt) was distributed diffusely throughout the cytoplasm and in the nucleus without accumulating at mitochondria (Figure 2, panels *D, H*). The significant amount of nuclear staining seen with this construct suggests that the Bcl-XL tail-anchor prevents nuclear import, perhaps because it is essential for the formation of Bcl-XL dimers [39] that unlike the monomer, are too large to diffuse through the nuclear pore. The localization of Bcl2-acta, Bcl2-cb5, BclX-acta, BclX-cb5 and BclX-Δtm detected by immunofluorescence was also confirmed by subcellular fractionation of cell lysates (data not shown).

### In MCF-7 cells Bcl-2 and Bcl-XL regulate organelle specific pathways of apoptosis differently

**Figure 3:**
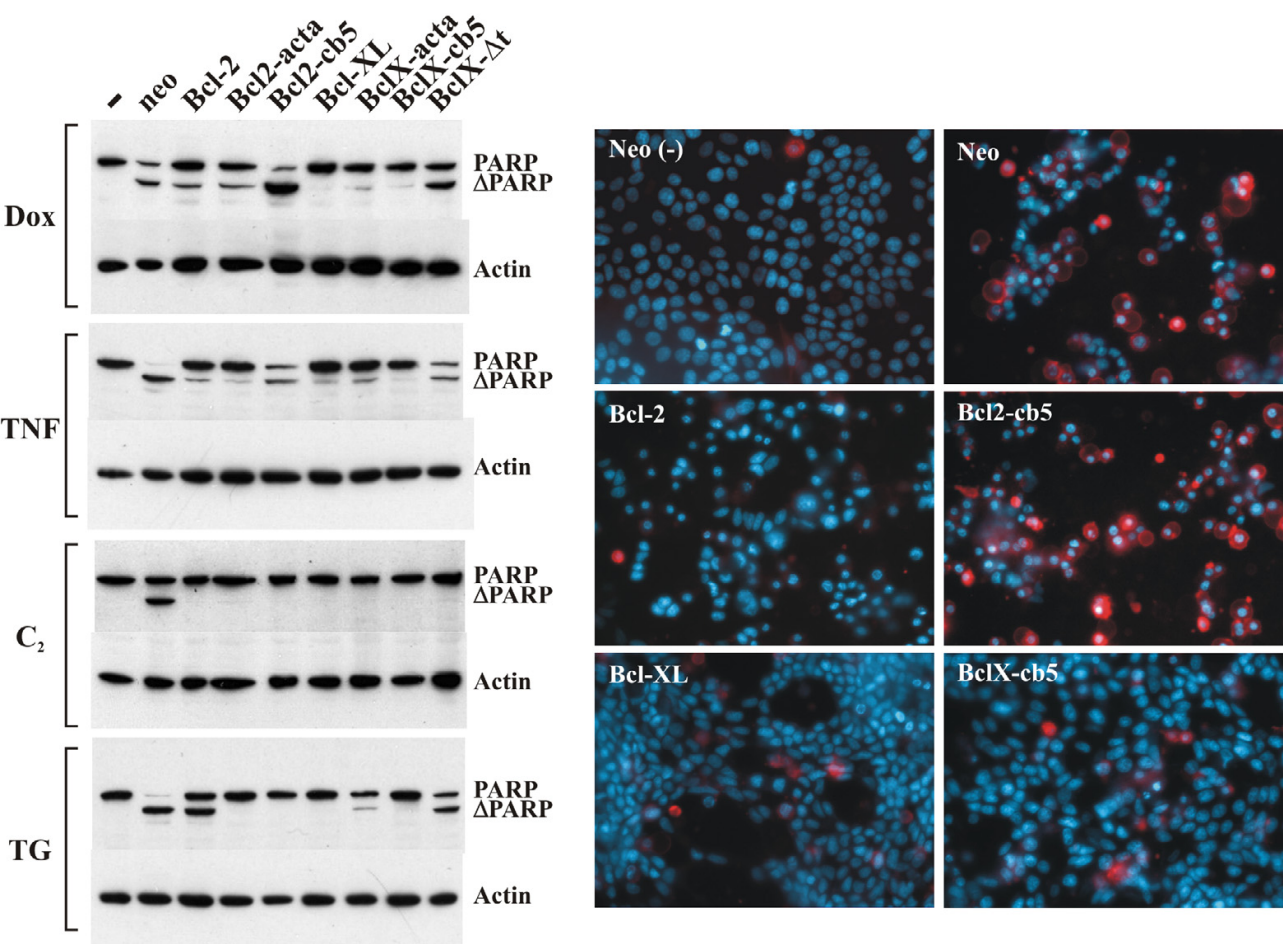
**Prevention of apoptosis in MCF-7 cells by Bcl-2 and Bcl-XL and by mutants with restricted subcellular localization. A) **Cell lysates containing 20 μg of protein from vector control (neo) or transfected MCF-7 cells expressing wild type proteins or proteins with restricted subcellular localization [ER (Bcl2-cb5, BclX-cb5), mitochondria (Bcl2-acta, BclX-acta) or nucleo-cytoplasm (BclX-Δt)] as indicated above the lanes were immunoblotted for PARP and actin. Cells were exposed to doxorubicin (Dox), TNFα and cycloheximide (TNF), ceramide (C_2_), or thapsigargin (TG) as in Figure 1. Untreated vector transfected cells (-). Expression of Bcl-2, Bcl-XL and the targeted mutants does not change the amount of PARP in untreated cells. The migration position of the 89 kDa caspase cleaved PARP fragment is indicated as ΔPARP. **B) **MCF-7 cells either untreated and transfected with the control plasmid (Neo -) or stably expressing the indicated protein and treated with TNFα and cycloheximide were stained with Hoescht 33342 (blue) or Annexin V coupled to Alexa Fluor594 (red) and visualized by epifluorescence microscopy. The width of each image is ~660 μm.

Using MCF-7 clones expressing Bcl-2, Bcl-XL or targeted mutants, we assessed the differences in inhibition of apoptosis induced by the agents characterized above. The dose and time were selected such that most of the PARP in the vector expressing control cells is cleaved to the characteristic 89 kDa fragment (ΔPARP). This dose was chosen because the closer the treatment is to the limit of inhibition, the more sensitive the assay is to differences between mutants. As expected, in cells expressing Bcl-2 or Bcl2-acta there was much less PARP cleavage. However, Bcl2-cb5 is largely ineffective at preventing doxorubicin induced cleavage of PARP. To our surprise, BclX-cb5 functioned differently than Bcl2-cb5 and prevented PARP cleavage as effectively as Bcl-XL and BclX-acta. Although BclX-Δtm was less effective than Bcl-XL it was much more effective at preventing PARP cleavage than Bcl2-cb5. Similarly, there was little to no inhibition of TNFα induced PARP cleavage by Bcl2-cb5 (Figure 3). In contrast, BclX-cb5 was relatively effective at inhibiting apoptosis induced by TNFα. This suggests that there is a fundamental difference in the function of Bcl-2 and Bcl-XL at the endoplasmic reticulum. Although based on this data alone it is formally possible that Bcl2-cb5 is misfolded and non-functional the fact that Bcl2-cb5 prevents apoptosis induced by either ceramide or TG demonstrated that Bcl2-cb5 is a functional protein (Figure 3).

When apoptosis was elicited by ceramide or TG, both Bcl2-cb5 and BclX-cb5 were as, or more, effective than the respective wild type proteins and mitochondrial targeted mutants (Figure 3). Ceramide treatment that resulted in cleavage of all of the PARP in control cells was not inhibited very well by any of the proteins, so for this agonist a concentration was used that resulted in cleavage of about half the PARP in the control cells (neo). For TG treated control cells, all the PARP was cleaved and/or degraded, and the amount of PARP cleavage in Bcl-2, BclX-acta and BclX-Δt cells indicates that this dose and time of TG treatment is close to the maximum inhibited by these proteins. As Bcl-2 expression is approximately four times higher than Bcl-XL and protection from apoptosis is similar but incomplete, Bcl-XL is at least four times as efficient as Bcl-2 at inhibiting cell death due to TG.

The striking difference in activity of Bcl2-cb5 and BclX-cb5 was also evident when TNFα induced apoptosis was assessed qualitatively by staining cells with Hoescht dye (to visualize chromatin condensation) and Annexin V (to visualize externalization of phosphatidylserine at the plasma membrane) (Figure 3B). Staining cannot be used to assess cells treated with doxorubicin as the drug is highly fluorescent. Moreover in MCF-7 cells treated with TNFα, quantification is difficult because the period and extent of chromatin condensation is small and it is followed by extensive chromatin degradation resulting in loss of staining. Nevertheless, it is qualitatively obvious from the images that BclX-cb5 inhibits apoptosis induced by TNFα while Bcl2-cb5 does not.

Thus, targeting Bcl-2 and Bcl-XL to endoplasmic reticulum has drastically different effects on function, depending on the agent used to elicit apoptosis. In contrast, mitochondrial localized Bcl2-acta and BclX-acta were very effective against all four agents, demonstrating that the mitochondria are a point of convergence in the apoptosis pathways induced by a wide variety of drugs (Figure 3A).

Surprisingly, BclX-Δt was considerably more effective than vector control in preventing apoptosis due to all three agents (Figure 3A). This suggests that at least a part of the protective ability of Bcl-XL does not require membrane binding mediated by a tail-anchor. A significant proportion of BclX-Δt measured by quantitative immunoblotting was located in the nucleus by immunofluorescence microscopy (Figure 2, panels *D *and *H*) where the relevant binding partners that regulate apoptosis are probably not accessible. Therefore, our data may underestimate the intrinsic activity of BclX-Δt in prevention of apoptosis. We were not able to systematically examine cells expressing Bcl2-Δt to determine the requirement for membrane insertion of this protein because immunoblotting of cells expressing Bcl2-Δt indicated more than half of the molecules were modified as detected by decreased mobility on SDS-PAGE (data not shown). Because this modification has an unknown effect on function, Bcl2-Δt was not examined further.

### In rat fibroblasts Bcl-2 and Bcl-XL regulate organelle specific pathways of apoptosis differently

**Figure 4:**
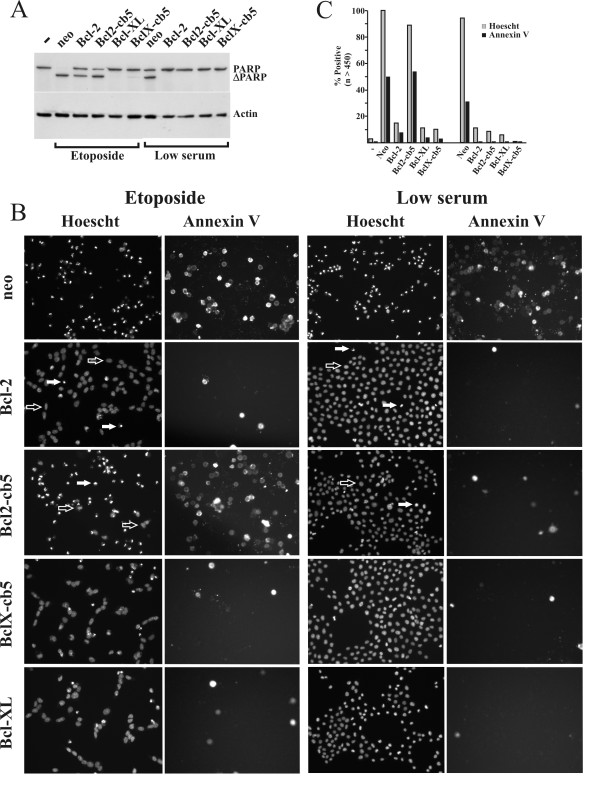
**Prevention of apoptosis in Rat-1 cells by Bcl-2 and Bcl-XL and by ER localized mutants. A) **Cell lysates containing 20 μg of protein from vector control (neo) or transfected Rat-1 cells expressing wild type proteins or ER localized mutants (Bcl2-cb5, BclX-cb5) as indicated above the lanes were immunoblotted for PARP and actin. Cells were exposed to etoposide or low serum. Untreated vector transfected cells (-). The migration position of the 89 kDa caspase cleaved PARP fragment is indicated as ΔPARP. **B) **Rat-1 cells transfected with the control plasmid (Neo -) or stably expressing the indicated protein and treated with TNFα and cycloheximide were stained with Hoescht 33342 or Annexin V coupled to Alexa Fluor594 (as indicated above the panels) and visualized by epifluorescence microscopy. A few normal nuclei are indicated by open arrowheads while examples of condensed nuclei are indicated by solid arrowheads. The width of each image is ~660 μm. **C) **Quantification of the results from experiments similar to those shown in **B)**. Each determination represents 450–600 cells analyzed converted to % positive by dividing by the total number of Hoescht stained cells, independent of whether chromatin condensation was observed.

To determine if the difference between Bcl2-cb5 and BclX-cb5 is specific to MCF-7 cells we expressed Bcl-2, Bcl-XL, Bcl2-cb5 and BclX-cb5 in rat fibroblasts. We have shown previously that Bcl2-cb5 prevents apoptosis induced by agents that target the ER but does not inhibit apoptosis initiated at mitochondria in Rat-1 cells [29,30]. Based on our earlier results we compared cell death induced by etoposide and low serum. Because we are comparing only these two agents it is also possible to use other assays in addition to PARP cleavage to measure the induction of apoptosis in the Rat-1 cell clones. As expected, control cells transfected with the empty vector (neo) are very sensitive to both etoposide and low serum (Figure 4), as a result PARP is cleaved, the nuclei condense as shown by Hoescht staining, and lipid asymmetry at the plasma membrane is lost resulting in externalization of phosphatidylserine (detected by labelling cells with Annexin V). In Rat-1 cells nuclear condensation is dramatic and quantitative thus, the results can be quantified from micrographs (Figure 4C). Bcl-2, Bcl-XL and BclX-cb5 all inhibited, but did not completely eliminate, nuclear condensation. Bcl2-cb5 was not effective at inhibiting etoposide induced nuclear condensation. Similarly, the fraction of cells that are annexin V positive is similar for the controls and Bcl2-cb5 expressing cells, whereas it is much reduced for cells expressing Bcl-2, Bcl-XL and importantly BclX-cb5 (Figure 4B–C). The most likely explanation for this result is that similar to MCF-7 cells, ER localized Bcl2-cb5 is unable to inhibit etoposide induced apoptosis even though it can be inhibited by BclX-cb5 proteins located at the same organelle. To demonstrate that this is a bona fide difference in activity between the two proteins and not a result of Bcl2-cb5 being inactive in rat fibroblasts, apoptosis was also induced in the cells by growth in low serum. Under these treatment conditions both Bcl2-cb5 and BclX-cb5 prevent apoptosis as well as the wild type proteins (Figure 4). Although all of the Bcl-2 family proteins prevent nuclear condensation as measured by Hoescht staining, none of the proteins prevented shrinkage of the nucleus (Figure 4) that occurs when cells are grown in low serum. While the difference in efficacy of BclX-cb5 and Bcl2-cb5 is obvious for these clones, these cells express unknown quantities of endogenous Bcl-2 and Bcl-XL complicating quantitative assessment.

### Bcl-XL is ten times more effective than Bcl-2 at preventing apoptosis in MCF-7 cells

It is clear from the results above that Bcl-2 and Bcl-XL inhibit many different forms of apoptosis. Previous reports suggest that expression levels of Bcl-2 or Bcl-XL in cells determine the extent of inhibition [40]. While there are some indications in the data above that Bcl-XL is more efficient than Bcl-2 at inhibiting apoptosis, quantitative comparison of Bcl-2 and Bcl-XL requires selection of transfected clones stably expressing known amounts of protein that does not vary with multiple passages, unlike what can be observed with pools of transfectants used in previous studies. As expected for stable clones of cell lines, all the cells expressed the transfected protein after expansion, as assessed by immunofluorescence (data not shown). Because our preliminary experiments demonstrated that Bcl-XL was more effective than Bcl-2 at preventing apoptosis, transfected cell lines grown in estrogen depleted medium (to decrease endogenous Bcl-2) were identified that consistently expressed roughly equipotent amounts of either Bcl-2 or Bcl-XL (range 1.2 to 4.5 ng /μg protein or 0.3 to 0.8 ng /μg protein, respectively and summarized in Table 1).

To accurately measure Bcl-2 and Bcl-XL in the cells we used quantitative immunoblotting. Standard curves on the same blots as the cell lysates demonstrated that detection of protein concentration was linear throughout the range needed for detection of the proteins. It was also essential to screen cells for equivalent expression after growth in estrogen depleted medium as a number of the clones exhibited striking changes in the amount of protein expressed in response to estrogen (data not shown). This is presumably a result of the site of integration of the exogenous DNA in the genome of the MCF-7 cells. Changes in the amount of the anti-apoptotic proteins during apoptosis were measured for all of the proteins, and with the exception of Bcl2-acta after doxorubicin treatment, paralleled each other and did not correlate with functional effects (see below). Furthermore, we extensively tested two separate clones that expressed closely matched levels of Bcl-2 (3.6 and 4.5 ng Bcl-2/μg protein) and performed partial comparisons for independent duplicates of the other cell lines. In all cases we obtained similar results, indicating that observed differences between the cell lines used for quantitative comparisons below were not due to clonal variation, but were due to the expression of the exogenous proteins (data not shown). Thus, we conclude that these clones can be used to compare quantitatively the effects of Bcl-2, Bcl-XL and the targeted mutants.

The assays chosen to measure apoptosis were cleavage of PARP and clonal survival. As shown above, PARP cleavage occurs downstream of convergence of the different apoptosis pathways and there is no feedback activation of caspase 8 after treatment of MCF7 cells with drugs (Figure 1). This lack of feedback amplification, likely due to lack of caspase 3, simplifies the interpretation of PARP cleavage data making it more useful for quantitative comparisons. Finally, PARP cleavage assayed by quantitative immunoblotting appears to have a larger dynamic range than either Hoescht staining or Annexin V labelling (data not shown).

**Figure 5:**
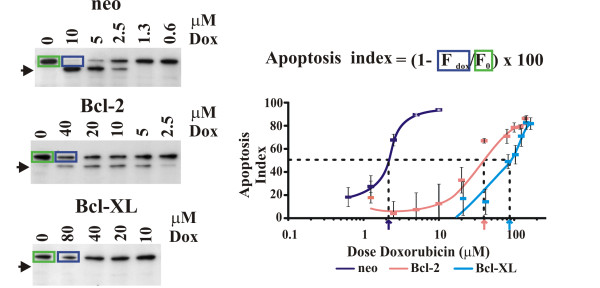
**Quantitative determination of PARP cleavage and degradation in MCF-7 cells following induction of apoptosis by treatment with doxorubicin. **The left panel shows the results of vector transfected (Neo) (top), Bcl-2 (middle) and Bcl-XL (bottom) expressing MCF-7 cells treated for 48 hours with increasing doses of doxorubicin as indicated, and cell lysates immunoblotted for PARP. The migration position of the 89 kDa PARP fragment diagnostic of caspase cleavage is indicated to the left of the blots (arrow). The antibody used for immunoblotting does not recognize cleavage products generated by other proteases activated during apoptosis. Therefore, both PARP cleavage and degradation were combined into a single apoptosis index (right panel, top) determined by measuring the band intensities for the116 kDa full length PARP in untreated cells (green box, 0), and in cells treated with doxorubicin (e.g. blue box) for the dose indicated above the lanes, on a Kodak image station. Actin blots were used to demonstrate equal protein loading in each lane (Figure 6). This index is plotted versus dose of doxorubicin for vector transfected cells (neo), and for cells expressing 3.6 ng Bcl-2 and 0.9 ng Bcl-XL per μg of total cell protein, respectively (right panel, bottom). EC_50 _values determined from the resulting dose response curves are indicated with coloured upward arrows.

A further qualitative difference between inhibition of apoptosis by Bcl-2 and Bcl-XL was evident at doses of doxorubicin that were effective at overcoming Bcl-2/Bcl-XL resistance. At these concentrations, cleavage of PARP at the site that generates the 89 kDa product characteristic of cleavage by Caspase 7 was evident in Bcl-2 expressing lines (Figure 5, left panels, arrow). By contrast, cleavage of PARP at this site was minimal in cells expressing Bcl-XL even at high doses (with the exception of cells expressing BclX-Δt, Figure 3). Instead, the *total *amount of PARP was decreased, indicating the degradation products in these cells were not recognized by the antibody (Figure 5, left panels). As many other proteases, including lysosomal proteases, are active during apoptosis [41], this suggests that Bcl-XL expression almost completely inhibits activation of effector caspases but not other proteases that mediate doxorubicin induced cell death. Since the 89 kDa product was eventually also degraded in Bcl-2 expressing cells, these additional proteases are active in clones expressing either protein. Degradation of PARP is still a specific indicator of programmed cell death and not a result of a general loss of cell protein as shown by blotting for actin (see below).

**Figure 6:**
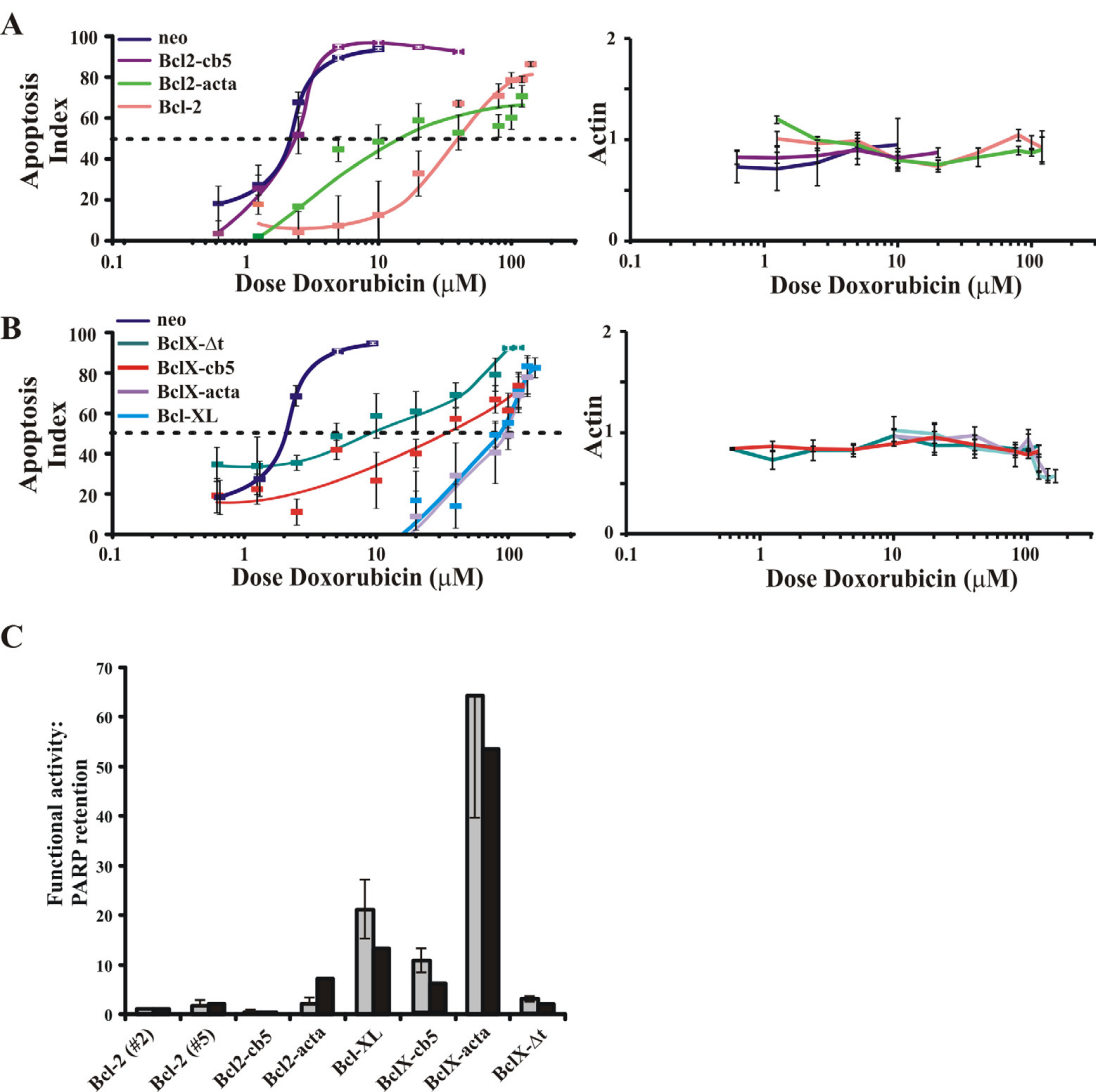
**The functional activity of Bcl-XL in preventing doxorubicin induced apoptosis is about ten times greater than that of Bcl-2. **Cell lines expressing **A**) Bcl-2, Bcl2-cb5 or Bcl2-acta and **B**) Bcl-XL, BclX-cb5, BclX-acta or BclX-Δt were treated with the specified dose of doxorubicin, and plotted against the apoptosis index (measured as in Figure 5). The data representing the dose response curves for vector transfected control cells (neo), Bcl-2 or Bcl-XL expressing cells shown in Figure 5 is duplicated here for comparison. The relative intensity recorded for actin blots prepared from the same samples are shown to the right and indicate that equivalent amounts of protein were analyzed for all samples. Bars indicate standard error of the mean, n ≥ 4.** C) **The EC_50 _values in μM doxorubicin for Bcl-2, Bcl-XL and mutants located at the ER (Bcl2-cb5, BclX-cb5), mitochondria (Bcl2-acta, BclX-acta) or cytoplasm (BclX-Δt) as determined from the graphs shown in **A**) and **B)**. To correct for expression of the individual proteins these values were divided by the concentration of anti-apoptotic protein (expressed as nMol/mg total cell protein normalized to Bcl-2 #2) in each cell line. Grey bars – based on expression levels prior to drug treatment. Black bars – based on expression levels after drug treatment at the EC50.

Loss of full length PARP is the most appropriate measure of cell death since both cleavage and degradation are accounted for (Figure 5, right panel). Using this metric as an apoptosis index we constructed doxorubicin dose response curves for control cells (neo) and cell clones expressing Bcl-2, Bcl2-cb5 or Bcl2-acta (Figure 6A) and for Bcl-XL, BclX-cb5, BclX-acta or BclX-Δt (Figure 6B) from immunoblots. This data confirms that for a wide range of drug concentrations the differences in inhibition of apoptosis noted with a single concentration of doxorubicin (Figure 3A) are preserved.

Comparison of the dose response curve for Bcl2-cb5 with the control demonstrates a complete lack of activity for Bcl2-cb5 at every concentration of doxorubicin tested (Figure 6A). To compare different cell lines directly, dose response curves were used to obtain an EC_50_, defined as the drug concentration that caused the disappearance and/or cleavage of half of the pre-treatment PARP (Figure 5 arrows, left panel).

The absolute amount of the anti-apoptotic proteins in each cell line and the apoptosis index were used to derive a measure of functional activity, defined as the EC_50 _of doxorubicin in μM (Figure 6A) divided by molar amount of anti-apoptotic protein expressed per μg of cell protein. Calculation of functional activity in this way permits normalization of the data for differences in the expression level of the exogenous proteins. However, such normalization is complicated by the fact that the amount of protein expressed is altered by drug treatment. Therefore, to determine the true extent of variation within the data and provide estimates of the relative efficacy of the different proteins, functional activities were calculated using both the amount of exogenous protein expressed prior to drug treatment and the amount of exogenous protein present at the time of assay at the EC50 for doxorubicin for each clone. The results of both calculations were compared by normalizing to Bcl-2 clone #2 which was arbitrarily set to 1). As a control to show the amount of variation for Bcl-2 we examined clone #5 as part of this analysis. Using this method to determine activity, it is clear that on a molar basis Bcl-XL prevented PARP degradation at a concentration of doxorubicin that is at least ten times higher than was inhibited by Bcl-2 (Figure 6C). Part of this increased efficacy may be due to the fact that the Bcl-XL associated with the ER is effective, whereas Bcl-2 is not able to prevent doxorubicin induced apoptosis from the ER. However, even when targeted to mitochondria where both proteins are active against doxorubicin induced apoptosis, Bcl-XL is much more efficacious than Bcl-2 (Figure 6C).

The shape of the dose response curve for Bcl2-acta differs from that of both wt Bcl-2, and BclX-acta (Figure 6A and 6B). Unlike the other mutants, addition of 5–20 μM doxorubicin strongly reduced the amount of Bcl2-acta as determined by quantitative immunoblotting. Because the same effect was seen in several different independently derived cell clones including those expressing more or less Bcl2-acta than the clone used here (data not shown) we presume that Bcl2-acta is more susceptible to a protease that is activated by low doses of doxorubicin. It is unclear whether this proteolytic activity is related to the organelle specific degradation of Bcl-2 that has been reported in other cell types after exposure to cytotoxic agents [42]. We did not observe any pro-apoptotic caspase cleavage products of Bcl-2 [43] or Bcl-XL [44] that have been described in other systems, possibly due lack of caspase 3 in MCF-7 cells.

Dose response curves were also used to assess the requirement of membrane binding for Bcl-XL activity. In many cell types a substantial fraction of Bcl-XL is cytoplasmic (e.g. 17), or as in these MCF-7 cells, peripherally bound to membranes when cells are not stressed (Zhu, Leber and Andrews, unpublished data). BclX-Δt remains nucleo-cytoplasmic during apoptosis but when assayed with doxorubicin it has higher functional activity than Bcl-2 (Figure 6C). Nevertheless, this activity is much less than that of Bcl-XL. BclXL-cb5 is also membrane bound and is substantially more active than BclXL-Δt. However, we cannot be sure how much of the activity of the latter is under-estimated by mis-targeting to putatively inactive sites in nucleus, as noted above (Figure 3, panels *D *and *H*). Thus, a significant amount of the activity of BclX-cb5 may not be due to specific location at the ER and interaction with resident membrane proteins at that organelle, but rather by sequestering and inactivating cytoplasmic pro-apoptotic proteins. A similar mechanism has been proposed for the activity of Bcl2-cb5 in preventing apoptosis by sequestering Bad [21].

**Figure 7:**
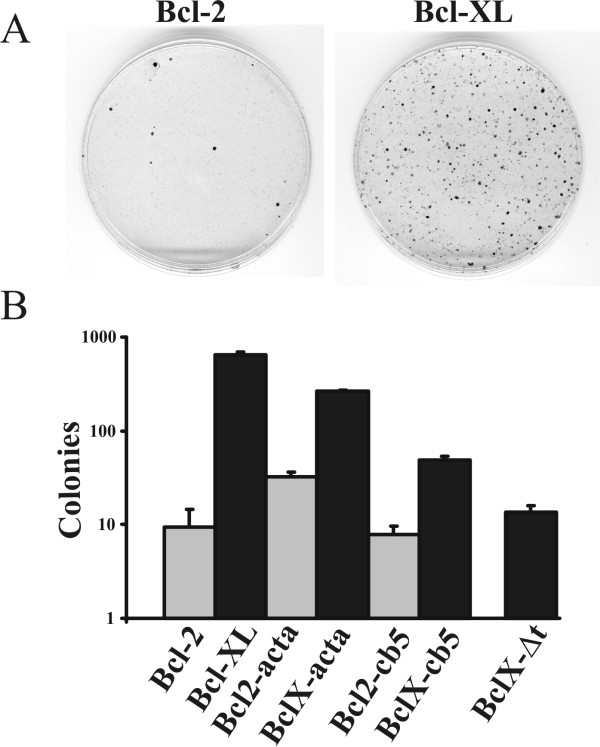
**The functional activity of Bcl-XL is more than ten times greater than Bcl-2 in increasing clonal survival after exposure to doxorubicin. A) **Cells expressing Bcl-2 or Bcl-XL, as indicated, were exposed to 5 μM doxorubicin in estrogen free medium for 1 hour, then replated in drug free, estrogen replete medium and allowed to grow for 14 days. Surviving colonies were visualized by staining with crystal violet. B) The number of surviving clones was counted and the results expressed as the number of surviving clones corrected for expression level of the respective protein for each cell clone. The data shown on a logarithmic scale is from one experiment performed in triplicate and is representative of three independent experiments. Bars indicate standard error of the mean.

As an independent and more clinically relevant means of comparing the effects of Bcl-2 and Bcl-XL on apoptosis [45], we measured clonal survival by re-plating efficiency of MCF7 cells exposed to 5μM doxorubicin for 1 hour [33]. This assay allows us to determine if the different effects on PARP cleavage and Annexin V labelling translate to the biologically and clinically relevant outcome of cell survival [37]. Consistent with results observed with measurement of residual PARP after exposure to doxorubicin, Bcl-XL was much more effective than Bcl-2 in promoting cell survival (Figure 7A). Furthermore, when corrected for expression level, the targeted mutants of Bcl-XL exhibited a similar order of efficacy, with BclX-acta > BclX-cb5 > BclX-Δt, although Bcl-XL was more effective than BclX-acta (Figure 7B). To our surprise Bcl2-cb5 promoted clonal survival after exposure of cells to 5 μM Doxorubicin for one hour even though it was unable to prevent cleavage of PARP after exposure to the same amount of drug for 24 hours. Therefore, the replating assay detected residual activity of Bcl2-cb5 not detectable with the PARP cleavage assay. However, the rest of the clonal survival data parallels that observed for cleavage of PARP.

## Discussion

Our results indicate that Bcl-XL and Bcl-2 are not functionally interchangeable. The organelle targeted mutants demonstrate that in both MCF-7 and Rat-1 cells Bcl-2 and Bcl-XL are *qualitatively *different: BclX-cb5 prevents apoptosis due to TNFα, doxorubicin (Figures 3, 6) and etoposide (Figure 4), whereas Bcl2-cb5 is ineffective. This extends our concept of organelle specific pathways of apoptosis by demonstrating that *within a single cell type*, a stressful stimulus elicits events at the ER differentially inhibited by Bcl-XL compared to Bcl-2. As apoptosis can be inhibited at either ER or mitochondria, this implies that changes at both organelles are necessary for apoptosis [22]. The most likely reason that when corrected for expression targeted mutants appear more effective than wild-type proteins is that at equal levels of expression there is twice as much protein at a single organelle. We speculate that for this reason the targeted mutants more efficiently inhibit one essential pathway whereas the wild-type proteins partially inhibit two pathways (Figure 6C).

The difference in efficacy we observed for BclX-cb5 and BclX-acta suggests that inhibition of apoptosis by sequestration of cytoplasmic effectors (the presumed mechanism of action for BclX-cb5) may be an activity independent from another function of the Bcl-XL that integrates into mitochondria. It is likely that this latter activity involves inhibiting oligomerization of Bax and Bak [51].

We also noted a *quantitative *difference between Bcl-2 and Bcl-XL in preventing doxorubicin induced apoptosis: Bcl-XL is about ten times more efficient than Bcl-2. This marked difference may explain the different prognostic values of Bcl-2 compared to Bcl-XL expression in human breast cancer samples independent of the presence of the estrogen receptor.

Our experiments demonstrate that small differences in the amount of anti-apoptosis proteins lead to large differences in cell survival. This is especially notable with Bcl-XL, and has important implications for clinical correlative studies where the ability to distinguish between different levels of expression of Bcl-XL and other anti-apoptotic proteins by immunohistochemistry is limited, and in which intercellular variability in the expression of Bcl-XL is within this critical range. While important clinical correlations have been found in many studies, our results suggest that biologically relevant variation may not always be detectable by these assays.

Because anti-apoptotic Bcl-2 family proteins participate in complex pathways with many components, the qualitative and quantitative differences in function for Bcl-2 and Bcl-XL may be mediated by altered binding affinity to other proteins that affect apoptosis (including but not limited to pro-apoptotic Bcl-2 family members, as has been recently reported, 46). Membrane binding is also important for many aspects of the function of these proteins, including the binding of pro-apoptotic Bcl-2 family proteins [47,48]. Therefore, the mechanisms and consequences of membrane integration of different anti-apoptotic proteins [39,46,49-51] is an important component in elucidating the differences between family members. Our results suggest that efficient sequestration of pro-apoptotic proteins depends on both the intrinsic preferences of Bcl-2 and Bcl-XL for particular targets as well as which organelle membrane the proteins are associated with. The most important general implication of our findings however, is that the exact mechanism of action in preventing apoptosis must be determined independently and specifically for each Bcl-2 family protein.

## Conclusion

When examined in the same cellular context, Bcl-2 and Bcl-XL differ substantially in the potency with which they inhibit apoptosis, mediated in part by differences in the inhibition of specific subcellular pathways.

## Competing interests

The author(s) declare that they have no competing interests.

## Authors' contributions

AF carried out the cell transfections of MCF-7 cells with plasmids encoding Bcl-XL or the targeted mutants, clonal selection, quantitative immunoblotting for the PARP assays, and contributed to the analysis and interpretation of data. WZ did the immunofluorescence/subcellular localization experiments, all experiments with the Rat-1 cells, Hoescht staining and Annexin V labeling experiments, qualitative western blots for PARP cleavage used to compare different drugs and the clonal survival assays. CH designed and constructed the plasmids encoding the Bcl-XL subcellular mutants. BL and DWA conceived, designed and directed the study, contributed to the analysis and interpretation of data, and drafted the manuscript. All authors read and approved the final manuscript.

## Pre-publication history

The pre-publication history for this paper can be accessed here:



## Figures and Tables

**Table 1:** Expression of Bcl-2, Bcl-XL and mutant proteins in MCF-7 cells.

**Cell Clone**	**Number of Independent Experiments**	**Expression of Bcl-2 or Bcl-XL proteins (ng/μg total protein) no drug**	**Expression of Bcl-2 or Bcl-XL proteins (ng/μg total protein) at the EC50 dose of doxorubicin**	**EC 50 doxorubicin (μM)** ** Inhibition of PARP degradation.**
Neo	5	n.d.	n.d.	1.9 ± 0
Bcl-2 (#2)	6	4.5 ± 1.0	2.5 ± 0.1	22.8 ± 3.3
Bcl-2 (#5)	7	3.6 ± 0.7	1.6 ± 0.1	30.6 ± 1.0
Bcl2-acta (#24)	7	1.2 ± 0.3	0.2 ± 0.02	13.2 ± 1.7
Bcl2-cb5 (#18)	5	1.5 ± 0.4	1.3 ± 0.02	2.3 ± 0.1
Bcl-XL (#42)	6	0.8 ± 0.2	0.7 ± 0.05	85.4 ± 0.6
BclX-Δt (#1)	5	0.6 ± 0.1	0.5 ± 0.01	8.7 ± 1.7
BclX-acta (#6)	4	0.3 ± 0.1	0.2 ± 0.02	97.8 ± 1.5
BclX-cb5 (#24)	5	0.6 ± 0.1	0.6 ± 0.1	32.3 ± 1.8
